# Balancing demands and rewards: a cross-sectional examination of well-being and professional identity among nurses

**DOI:** 10.1177/17449871261426973

**Published:** 2026-04-09

**Authors:** Cicilia Nagel, Kerstin Nilsson

**Affiliations:** Senior Lecturer, Department of Health Sciences, Kristianstad University, Kristianstad, Sweden; Professor, Department of Health Sciences, Kristianstad University, Kristianstad, Sweden; Department of Laboratory Medicine, Lund University, Lund, Sweden

**Keywords:** descriptive quantitative, health promotion, nurses, survey, statistical analysis, workforce and employment

## Abstract

**Background:**

Increasing healthcare demands and a growing nursing shortage contribute to high workloads, psychosocial strain, and risks to care quality. Organisational conditions, job demands, and resources strongly influence nurses’ well-being, retention, and performance.

**Aim:**

To examine how nurses balance organisational demands and opportunities, and how these factors relate to work environment, well-being, and professional identity.

**Method:**

A cross-sectional survey was completed by 2704 nurses in Sweden. Participants were grouped by age based on the median (⩽51 vs ⩾52 years) and by gender. Descriptive statistics and chi-square tests were conducted, with missing data handled using pairwise deletion. Findings were interpreted through the job demands–resources and effort–reward imbalance frameworks.

**Results:**

Younger nurses experienced higher physical workloads, lower recognition, lower joy and satisfaction, whereas older nurses reported better health, higher psychological work ability, and greater professional pride. Female nurses reported stronger self-imposed pressure and social community, whereas male nurses indicated higher work ability and better sleep. Across all groups, nurses demonstrated strong professional identity, but workload and recovery burdens were evident.

**Conclusion:**

Strengthening key resources – autonomy, recognition, and social support – while addressing systemic workload pressures may enhance sustainability, particularly for early-career nurses and support long-term health, professional identity, and workforce retention.

## Background

Demographic shifts, including population aging and declining youth cohorts, have intensified the demand for healthcare services across Europe (Bernini et al., 2024; OECD/European Commission, 2024). Concurrently, a persistent global shortage of nurses – further aggravated by the COVID-19 pandemic – has placed unprecedented strain on healthcare systems and amplified mental health challenges among nursing staff (WHO, 2020). Nurses’ work is often characterised by high demands, staffing shortages, and increasing complexity of care – conditions that are strongly associated with stress, burnout, and turnover, raising concerns about both workforce sustainability and quality of patient care (Kelly et al., 2021; WHO, 2020). A previous study demonstrated that the provision of fundamental care requires nurses to exhibit compassion, empathy, and relationship-building skills, as well as the ability to extend their efforts beyond routine practice during emergencies (Kitson et al., 2022). Numerous studies have reported a high prevalence of stress, anxiety and depression, and burnout (Kiptulon et al., 2024; Nagel et al., 2022; Nagel and Nilsson, 2022). Long working hours and insufficient organisational support have been identified as key factors contributing to these adverse outcomes (Michie and Williams, 2003; Nagel et al., 2022). In addition, workload and work pressure negatively affect job performance, increasing the risk of medical errors, reducing productivity, and compromising quality of care (Hämmig, 2018). The nursing work environment is therefore a critical determinant of healthcare quality, as it influences nurses’ job satisfaction and retention, patient outcomes, and organisational performance (Lake et al., 2019; Nascimento and Jesus, 2020).

A growing body of research emphasises that psychological well-being and workplace conditions are central to nurses’ retention (Altalhi and Alilyyani, 2025). Research on health in working life has long emphasised that sustainable employability and well-being are shaped by the interaction between individual, organisational, and societal factors. The field of work science provides a broader framework for understanding how job design, leadership, participation, and learning conditions affect employees’ health, motivation, and long-term employability (Nilsson, 2020a). The sustainable working life for all ages (SwAge) model consists of four spheres for action and long-term employability of individuals: (1) health effects of the work environment, (2) personal finances, (3) social relations, support and sense of community, and (4) execution of work tasks (Nilsson, 2020b). These domains emphasise that sustainable working conditions are not only dependent on the absence of ill health but also on the presence of supportive structures that enable development, balance, and recovery across the life course. Integrating insights from work science and nursing research thus allows for a more comprehensive understanding of how organisational and psychosocial factors interact to influence nurses’ health, professional identity, and retention. Organisational factors such as workload, staffing levels, and managerial support shape the work environment (Nilsson and Nilsson, 2021). Drawing on the job demand–resource (JDR; Bakker et al., 2023) and effort–reward imbalance (ERI; Siegrist, 1996) frameworks, these dynamics can be understood as interactions between job demands, available resources, and perceived balance between effort and reward, all of which influence nurses’ motivation, health, and retention. Additionally, professional identity – and the experience of meaning and pride in work – constitutes a valuable resource for sustaining motivation and resilience (Kanwal et al., 2020). A supportive work environment has consistently been linked to improved patient safety, higher job satisfaction, and reduced burnout (Lucas et al., 2025). However, previous research has often examined work environment and professional identity separately, with less attention to how nurses themselves experience and navigate the balance between organisational demands, professional opportunities, and daily prioritisations. Research also indicates that nurses’ experiences of workload, control, and social support may differ by gender and age (Dziedzic et al., 2025; Lim et al., 2022). Addressing these aspects is essential to better understand the consequences for both the work environment and professional identity and to inform strategies that promote sustainable nursing practice. The aim of this study is therefore to explore how nurses experience and manage the balance between professional opportunities, organisational demands, and necessary prioritisations in their daily work and to examine the consequences for their work environment and professional identity.

## Research questions

1. How do nurses perceive and manage the balance between professional opportunities, organisational demands, and necessary prioritisations in their everyday practice?
2. What are the perceived consequences of this balance for nurses’ work environment and professional identity?
3. Are there differences in these experiences between younger and older nurses?
4. Are there differences in these experiences between male and female nurses?

## Method

### Design

This study used a quantitative, explorative cross-sectional design to capture nurses’ experiences of balancing professional opportunities, organisational demands, and daily prioritisations. The design allows examination of relationships between work demands, resources, and outcomes, as well as comparisons across age and gender. Findings are interpreted using the JD-R and ERI models to understand how work conditions and recognition relate to well-being and professional identity. Although causal conclusions cannot be drawn, the design provides a valuable snapshot to inform future longitudinal or intervention studies.

### Study setting and data collection

Data for the present study were collected via an online questionnaire distributed to registered nurses (*n* = 6829) in the south of Sweden, of which 2704 responded (response rate 40%). Data collection took place between April and June 2024. Participants worked across a range of hospitals and healthcare settings in the region, including general wards, specialised departments, ambulance services, and call centres, representing a variety of clinical roles and specialties. Participants were categorised into two age groups based on the sample median age (51 years), resulting in a younger age group (27–51 years) and an older age group (52–74 years). The median age was relatively high due to a larger proportion of respondents being 50 years or older, making the median an appropriate and data-driven cut-off for grouping. Using the sample median as the threshold ensured two approximately equal-sized groups, facilitated age-related comparisons and provided an empirically derived cut-off that reflected the age distribution of the responding cohort. Gender was recorded in three categories: male, female, and other. As only one participant identified as ‘other’, this category was excluded from statistical comparisons but is presented in the descriptive statistics. Descriptive statistics were used to summarise sample characteristics.

### Measures

The questionnaire used for measurements in this study was based on the SwAge model (Nilsson, 2020b, 2024), a theory-driven framework that integrates life-course perspectives on ageing with occupational health research and empirical studies of work environments. The model aligns conceptually with the JD-R and ERI models, emphasising how work demands, available resources, recognition, and rewards interact with employees’ health, competencies, and motivation to sustain working life. The SwAge model conceptualises employability and nine impact and determinant areas influencing individuals’ ability and willingness to remain in working life. These areas are organised into four spheres of employability: (1) health effects of work environments, including self-rated health and functional status, physical and mental work environments and working hours, work pace, and recovery; (2) financial incentives, reflecting personal financial situation and its implications for employability and work participation; (3) relationships, social support and participation, covering the role of the social environment both outside and inside work, including leadership, group dynamics, and collegial support; and (4) execution of work tasks and activities, referring to opportunities for motivation, appreciation and meaningfulness at work, as well as skills, knowledge, and competence development. For the present study, the focus was primarily on areas (1), (3), and (4), whereas financial incentives (2) were not investigated. Although the SwAge model provides a comprehensive conceptual framework, it is not a psychometrically validated measurement instrument. Survey items were designed to capture key domains of the model and were adapted from validated instruments used in health research and from previous waves of the SwAge study, demonstrating good construct validity. Responses were recorded on a 4-point Likert scale (completely agree, somewhat agree, somewhat disagree, completely disagree), which were dichotomised for analysis (agree–disagree) and categorical variables were created for group comparisons.

### Data analysis

Differences between age and gender groups in relation to the study variables were analysed using chi-square tests. Missing data were handled using pairwise deletion. Statistical significance was set at *p* < 0.05, and analyses were conducted using IBM SPSS Statistics, version 27. As the analyses were exploratory and aimed at identifying patterns rather than testing predefined hypotheses, no formal adjustment for multiple comparisons was applied; findings should therefore be interpreted with caution (Bender and Lange, 2001; Perneger, 1998).

### Ethical considerations

The study was conducted in accordance with the principles of the Declaration of Helsinki (World Medical Association [WMA], 2024) and approved by the Swedish Ethical Review Authority (Ref. No. 2023-04931-02). Participation was voluntary, and information about the study purpose, procedures, and confidentiality was provided on the first page of the online questionnaire. Informed consent was obtained through participants’ decision to complete and submit the survey. Data were anonymised prior to analysis and stored securely to prevent unauthorised access. This study is reported in accordance with the Strengthening the Reporting of Observational Studies in Epidemiology (STROBE) (Von Elm et al., 2007) guidelines for cross-sectional studies (checklist available as Supplemental Material).

## Results

A total of 2704 registered nurses (including specialist nurses) participated in the study, with a mean age of 51 years (range 27–74). The majority were women (90%), born in Sweden (89%) and living with a partner (77%). About 43.6% of the participants stated that they did not have children living at home. Most nurses had long professional experience, with more than two-thirds having worked in the profession for over 16 years. The majority were employed full-time (65%) and worked mainly day or evening shifts (70%). More than half of the participants reported short-term sickness absence (1–14 days) during the past 12 months. Descriptive characteristics of the sample are presented in Table 1.

**Table 1.** Descriptive statistics of the participants in this study.

Age, Median (Min–Max) Younger age group, *n* (Min–Max) Older age group, *n* (Min–Max)	51 (27–74) 1349 (27–51) 1355 (52–74)
Gender, *n* (%)	
Female	2415 (90.2)
Male	262 (9.8)
Other	1
Missing value, *n*	26
Place of birth, *n* (%)	
Sweden	2391 (89.1)
In a Nordic country, not Sweden	53 (2.0)
In a European country	135 (5.0)
Outside of Europe	103 (3.8)
Missing value, *n*	22
Relationship status, *n* (%)	
Married/Co-inhabitant	2069 (76.9)
Living apart	142 (5.3)
Single	478 (17.8)
Missing value, *n*	15
Children living at home, *n*	
None, *n*	1179
Age 0–3	229
Age 4–6	347
Age 7–12	622
Age 13–15	426
Age 16–19	462
Worked as a nurse, *n* (%)	
<5 years	90 (3.3)
6–10 years	287 (10.7)
11–15 years	499 (18.5)
>16 years	1816 (67.5)
Missing value	12
Working percentage of employment, *n* (%)	
100	1744 (64.7)
80–99	621 (23.1)
60–79	200 (7.4)
40–59	72 (2.7)
20–39	25 (0.9)
<19%	32 (1.2)
Missing value	10
Working shift, *n* (%)	
Only during day/evening	1866 (69.5)
Only nights	148 (5.5)
Combination day/evening/night	502 (18.7)
Combination day/evening/on-call	119 (4.4)
Other shifts	50 (1.9)
Missing value	19
During the past 12 months, I have been absent from work due to sickness, *n* (%)	
0 days	639 (23.7)
1–14 days	1632 (60.6)
15–59 days	301 (11.2)
>60 days	122 (4.5)
Missing value	10

Table 2 presents comparisons of perceived work demands between the older and younger age groups of nurses, as well as between male and female nurses. Several significant group differences were observed. A smaller proportion of nurses in the older age group (30%) compared to the younger group (35%) agreed that their work contains many physically heavy tasks (*p* = 0.002). Similarly, female nurses (32%) seemed less likely than male nurses (37%) to report that their work involved physically heavy tasks (*p* = 0.05). In contrast, male nurses (67%) seemed significantly more likely than female nurses (57%) to report that their work involves many psychologically heavy tasks (*p* = 0.01), although no significant age-related differences were found for this item (*p* = 0.07). Nurses in the older age group were more likely to disagree that shorter work hours could increase their well-being (26% vs 18%, *p* < 0.001), indicating that nurses in the younger age group expressed a stronger desire for reduced working hours. Finally, a gender difference was observed for self-imposed pressure at work where female nurses (61%) were significantly more likely than male nurses (45%) to agree that they put too much pressure on themselves (*p* < 0.001). No significant differences between age groups were found for this item (*p* = 0.25). No other items – such as high work pace, task clustering, or staffing shortages – showed statistically significant differences by age or gender.

**Table 2.** Work demands. Chi-square tests, comparing the older age group (above median age, 52–74) of nurses (*n* = 1355) with the younger age group (below median age, 27–51) of nurses (*n* = 1349) and male (*n* = 262) versus female (*n* = 2415) nurses. Percentage is calculated on the actual number of respondents to each variable.

Statement		Older age group (52–74), n (%)	Younger age group (27–51), *n* (%)	*p*-Value	Male, *n* (%)	Female, *n* (%)	*p*-Value
My work contains many physically heavy work tasks	Disagree	945 (70)	868 (65)	.002*	165 (63)	1633 (68)	0.05*
	Agree	405 (30)	476 (35)		94 (37)	775 (32)	
My work contains many psychologically heavy work tasks	Disagree	579 (43)	552 (41)	.07	87 (33)	1037 (43)	0.01*
	Agree	767 (57)	787 (59)		175 (67)	1360 (57)	
The work pace in my daily work is too high	Disagree	872 (65)	917 (68)	.14	186 (72)	1589 (66)	0.48
	Agree	474 (35)	429 (32)		73 (28)	817 (34)	
Work tasks usually cluster together to the point that I get frustrated	Disagree	838 (62)	831 (62)	.85	173 (66)	1482 (62)	0.11
	Agree	511 (38)	515 (38)		88 (34)	925 (38)	
Not having enough staff affects my ability to perform my work	Disagree	848 (63)	781 (58)	.06	168 (64)	1449 (60)	0.51
	Agree	501 (37)	562 (42)		94 (36)	954 (40)	
Shorter work hours could increase my well-being	Disagree	343 (26)	235 (18)	<.001*	64 (25)	508 (21)	0.28
	Agree	999 (74)	1106 (82)		196 (75)	1888 (79)	
I put too much pressure on myself in my work	Disagree	552 (41)	539 (40)	.25	144 (55)	938 (39)	<0.001*
	Agree	797 (59)	807 (60)		118 (45)	1468 (61)	

* χ²-test was used to compare. *p* < 0.05 is marked with *.

Table 3 presents comparisons of perceived work control between the older and younger age group of nurses, as well as between male and female nurses. Significant group differences were observed only in relation to the statement ‘*I get enough opportunities to utilise my skills and knowledge at work’.* A significantly larger proportion of nurses in the older age group (90%) than those in the younger age group (86%) agreed with this statement (*p* < 0.001). Similarly, a greater proportion of female nurses (88%) than male nurses (82%) reported having sufficient opportunities to utilise their skills and knowledge (*p* = 0.001). No significant age or gender differences were found for the remaining items. The proportions of nurses who agreed that they had time to perform planned work tasks, opportunities to take breaks, and opportunities for competence development were relatively similar across all groups.

**Table 3.** Work control. Chi-square tests, comparing the older age group (above median age, 52–74) of nurses (*n* = 1355) with the younger age group (below median age, 27–51) of nurses (*n* = 1349) and male (*n* = 262) versus female (*n* = 2415) nurses. Percentage is calculated on the actual number of respondents to each variable.

Statement		Older age group (52–74), *n* (%)	Younger age group (27–51), *n* (%)	*p*-Value	Male, *n* (%)	Female, *n* (%)	*p*-Value
I get enough opportunities to utilise my skills and knowledge at work	Disagree	138 (10)	191 (14)	<0.001*	48 (18)	278 (12)	0.001*
	Agree	1207 (90)	1148 (86)		214 (82)	2117 (88)	
I have time to perform the work tasks I had planned throughout the day	Disagree	335 (25)	317 (24)	0.08	47 (18)	595 (25)	0.09
	Agree	1011 (75)	1024 (76)		215 (82)	1806 (75)	
I get enough opportunities to take breaks at work	Disagree	543 (40)	522 (39)	0.66	91 (35)	961 (40)	0.59
	Agree	810 (60)	822 (61)		171 (65)	1447 (60)	
I get enough opportunities to partake in competence development	Disagree	519 (38)	517 (39)	0.99	95 (37)	929 (39)	0.51
	Agree	816 (62)	826 (61)		164 (63)	1463 (61)	

* χ²-test was used to compare. *p* < 0.05 is marked with *.

Table 4 presents comparisons of perceived social support at work between the older and younger age groups of nurses, as well as between male and female nurses. Significant differences were found for two of the measured items. A significantly larger proportion of nurses in the younger age group (82%) compared with those in the older age group (73%) agreed with the statement ‘*The social community that exists in my workplace makes me want to stay’* (*p* < 0.001). Similarly, female nurses (78%) were more likely than male nurses (71%) to agree with this statement (*p* < 0.001). These findings indicate that nurses in the younger age group who were female reported a stronger sense of belonging and motivation derived from workplace social relations. Additionally, a significant gender difference was observed regarding the perceived importance of collegial support. Female nurses (94%) were slightly more likely than male nurses (92%) to agree that collegial support is important for being able to do a good job (*p* < 0.001). No significant age difference was found for this item (*p* = 0.18).

**Table 4.** Social support. Chi-square tests, comparing the older age group (above median age, 52–74) of nurses (*n* = 1355) with the younger age group (below median age, 27–51) of nurses (*n* = 1349) and male (*n* = 262) versus female (*n* = 2415) nurses. Percentage is calculated on the actual number of respondents to each variable.

Statement		Older age group (52–74), n (%)	Younger age group (27–51), *n* (%)	*p*-Value	Male, *n* (%)	Female, *n* (%)	*p*-Value
I get enough support from my closest manager	Disagree	387 (29)	407 (30)	0.36	73 (28)	715 (30)	0.76
	Agree	956 (71)	935 (70)		188 (72)	1682 (70)	
I get enough support from my co-workers	Disagree	129 (10)	109 (8)	0.11	25 (10)	212 (9)	0.91
	Agree	1213 (90)	1236 (92)		237 (90)	2187 (91)	
The social community that exists in my workplace makes me want to stay	Disagree	357 (27)	239 (18)	<0.001*	76 (29)	517 (22)	<0.001*
	Agree	987 (73)	1101 (82)		186 (71)	1878 (78)	
I feel bullied/ shut out from the social community at work	Disagree	1303 (97)	1310 (97)	0.83	249 (95)	2339 (97)	0.23
	Agree	43 (3)	36 (3)		13 (5)	64 (3)	
The collegial support is detriment for me being able to do a good job	Disagree	95 (7)	80 (6)	0.18	30 (8)	143 (6)	<0.001*
	Agree	1248 (93)	1262 (94)		232 (92)	2253 (94)	

* χ^2^-test was used to compare. *p* < 0.05 is marked with *.

There were no statistically significant differences between age or gender groups regarding support from managers, support from co-workers, or experiences of being bullied or socially excluded at work. The majority of respondents across all groups reported receiving sufficient support from both managers and colleagues and denied experiences of bullying or social exclusion.

Table 5 presents comparisons of nurses’ experiences of work joy, pride, and professional identity between the older and younger age groups, as well as between male and female nurses. Several statistically significant differences were observed. Compared with the younger age group of nurses, the older age group were more likely to agree that they receive the recognition they deserve for their work (66% vs 60%, *p* < 0.001), are satisfied with their daily work (88% vs 85%, *p* < 0.001), experience joy in their daily work (87% vs 84%, *p* = 0.001), find their work stimulating (90% vs 87%, *p* < 0.001), and are satisfied with their current work tasks (88% vs 83%, *p* < 0.001).

**Table 5.** Work joy, pride and professional identity. Chi-square tests, comparing the older age group (above median age, 52–74) of nurses (*n* = 1355) with the younger age group (below median age) of nurses (*n* = 1349) and male (*n* = 262) versus female (*n* = 2415) nurses. Percentage is calculated on the actual number of respondents to each variable.

Statement		Older age group (52–74), *n* (%)	Younger age group (27–51), *n* (%)	*p*-Value	Male, *n* (%)	Female, *n* (%)	*p*-Value
Work is important in my life	Disagree	201 (15)	164 (12)	0.15	46 (18)	316 (13)	0.04*
	Agree	1150 (85)	1184 (88)		215 (82)	2095 (87)	
I get the recognition I deserve considering the effort I put in and what I perform at work	Disagree	459 (34)	544 (40)	<0.001*	100 (38)	895 (37)	0.16
	Agree	884 (66)	800 (60)		160 (62)	1505 (63)	
Overall, I feel proud over my work	Disagree	53 (4)	70 (5)	<0.001*	24 (9)	97 (4)	<0.001*
	Agree	1294 (96)	1273 (95)		236 (91)	2307 (96)	
Overall, I feel satisfaction with my daily work	Disagree	165 (12)	206 (15)	<0.001*	40 (16)	332 (14)	0.005*
	Agree	1181 (88)	1131 (85)		208 (84)	2067 (86)	
I experience joy in my daily work	Disagree	181 (13)	210 (16)	0.001*	53 (20)	332 (14)	0.005*
	Agree	1162 (87)	1134 (84)		208 (80)	2067 (86)	
I feel that my daily work is meaningful	Disagree	69 (5)	103 (8)	0.003*	21 (8)	151 (6)	<0.001*
	Agree	1275 (95)	1236 (92)		242 (92)	2243 (94)	
I feel that my work is stimulating	Disagree	133 (10)	181 (13)	<0.001*	36 (14)	273 (11)	0.002*
	Agree	1209 (90)	1165 (87)		225 (86)	2128 (89)	
Overall, I am satisfied with my current work tasks	Disagree	164 (12)	228 (17)	<0.001*	46 (18)	340 (14)	0.04*
	Agree	1179 (88)	1114 (83)		216 (82)	2057 (86)	
Overall, I am satisfied with my current work situation	Disagree	289 (21)	279 (21)	0.01*	60 (23)	202 (77)	0.33
	Agree	1056 (79)	1067 (79)		202 (77)	1900 (79)	
Big changes in my work situation makes me want to leave	Disagree	1103 (82)	1114 (83)	0.11	208 (80)	1987 (83)	0.59
	Agree	243 (18)	227 (17)		51 (20)	414 (17)	

* χ²-test was used to compare. *p* < 0.05 is marked with *.

A high percentage of nurses in both the older and younger age group reported feeling satisfied with their current work situation (79%; *p* = .01). No age-related differences were observed regarding whether work was considered important in life or whether major work changes made nurses want to leave their position. Gender differences followed a similar pattern, with female nurses generally reporting more positive experiences than male nurses. Female nurses were more likely to state that work is important in their life (87% vs 82%, *p* = 0.04), they feel proud of their work (96% vs 91%, *p* < 0.001), they experience joy in their daily work (86% vs 80%, *p* = 0.005), they find their work meaningful (94% vs 92%, *p* < 0.001), their work is stimulating (89% vs 86%, *p* = 0.002), and they are satisfied with their current work tasks (86% vs 82%, *p* = 0.04). No significant gender differences were found regarding recognition, overall work situation, or reactions to major work changes.

Table 6 compares nurses’ self-rated health, work ability, rest, and well-being between the older and younger age groups of nurses, as well as between male and female nurses. Several significant age- and gender-related differences were identified. A significantly larger proportion of nurses in the older age group (75%) compared to those in the younger age group (70%) reported good overall health and well-being (*p* < 0.001). Nurses in the older age group also reported better psychological work ability (86% vs 81%, *p* < 0.001), more likely to agree that they get enough rest between work shifts (69% vs 61%, *p* < .001), and that they seldom feel emotional exhaustion (64% vs 57%, *p* < 0.001). At the same time, nurses in the older age group reported poorer sleep quality, being more likely than those in the younger age group to wake up during the night (62% vs 50%, *p* < 0.001) and to have difficulties falling back asleep (45% vs 37%, *p* < 0.001). Nurses in the older age group were also more likely to report having had a work-related injury or illness (24% vs 17%, *p* < 0.001) and have slightly less capacity to handle the physical demands of work (93% vs 96%, *p* < 0.001). Moreover, they wish to work at a slower pace (42% vs 35%, *p* = 0.002).

**Table 6.** Health and ability to work. Chi-square tests, comparing the older age group (above median age, 52–74) of nurses (*n* = 1355) with the younger age group (below median age, 27–51) of nurses (*n* = 1349) and male (*n* = 262) versus female (*n* = 2415) nurses. Percentage is calculated on the actual number of respondents to each variable.

Statement		Older age group (52-74), *n* (%)	Younger age group (27-51), n (%)	*p*-Value	Male, *n* (%)	Female, *n* (%)	*p*-Value
Currently, I feel that my health/well-being is good	Disagree	330 (25)	405 (30)	<0.001*	65 (25)	661 (28)	0.004*
	Agree	1014 (75)	932 (70)		197 (75)	1733 (72)	
My current work ability is good in relation to the physical demands required to perform my work	Disagree	193 (14)	179 (13)	0.46	32 (12)	339 (14)	0.02*
	Agree	1147 (86)	1152 (87)		230 (88)	2047 (86)	
My current work ability is good in relation to the psychological demands required to perform my work	Disagree	186 (14)	250 (19)	<0.001*	34 (13)	395 (17)	0.02*
	Agree	1154 (86)	1085 (81)		226 (87)	1994 (83)	
For the most part I can handle the physical demands of my work	Disagree	89 (7)	54 (4)	<0.001*	8 (3)	134 (6)	0.24
	Agree	1256 (93)	1288 (96)		254 (97)	2265 (94)	
My current work is too psychologically strenuous for my health	Disagree	1105 (82)	1113 (83)	<0.001*	223 (86)	1974 (82)	0.15
	Agree	236 (18)	230 (17)		37 (14)	423 (18)	
I get enough rest between my work shifts	Disagree	423 (31)	524 (39)	<0.001*	93 (36)	846 (35)	0.50
	Agree	926 (69)	818 (61)		168 (64)	1557 (65)	
I often wake up during the night	Disagree	504 (38)	674 (50)	<0.001*	140 (55)	1040 (43)	0.01*
	Agree	831 (62)	662 (50)		116 (45)	1358 (57)	
I have difficulties going back to sleep when I wake up during the night	Disagree	743 (55)	845 (63)	<0.001*	174 (66)	1403 (58)	0.02*
	Agree	605 (45)	502 (37)		88 (34)	1003 (42)	
I often feel emotional exhaustion	Disagree	869 (64)	772 (57)	<0.001*	172 (66)	1454 (60)	0.06
	Agree	482 (36)	571 (43)		87 (34)	954 (40)	
I seldom feel well-rested	Disagree	784 (58)	650 (49)	<0.001*	136 (52)	1297 (54)	0.32
	Agree	562 (42)	684 (51)		126 (48)	1104 (46)	
I have/have had an injury or illness that I feel is caused by my work to some degree	Disagree	1000 (76)	1104 (83)	<0.001*	221 (87)	1864 (79)	0.01*
	Agree	312 (24)	225 (17)		34 (13)	497 (21)	
I wish to work at a slower pace	Disagree	783 (58)	873 (65)	0.002*	165 (63)	1480 (62)	0.71
	Agree	563 (42)	473 (35)		96 (37)	925 (38)	

* χ²-test was used to compare. *p* < 0.05 is marked with *.

Significant gender differences were observed in several items. Male nurses were more likely to report good health (75% vs 72%, *p* = 0.004) and better physical and psychological work ability (88% vs 86%, *p* = 0.02 for both). Male nurses were also more likely than female nurses to report fewer awakenings during the night (55% vs 43%, *p* = 0.01) and fewer difficulties returning to sleep (66% vs 58%, *p* = 0.02). Conversely, female nurses more often reported having had an injury or illness they believed was caused by their work (21% vs 13%, *p* = 0.01). No gender differences were found in perceived rest between shifts, emotional exhaustion, or the wish to work at a slower pace.

## Discussion

This discussion addresses the study’s four research questions, examining how nurses perceive and manage the balance between organisational demands and professional opportunities, the implications for work environment and professional identity and differences across age and gender. Overall, the findings depict a complex work context in which high physical and psychological demands coexist with strong professional pride and meaning. Interpreted through the JD-R model (Bakker et al., 2023), the results suggest an imbalance between substantial demands and uneven access to resources, with important consequences for nurses’ well-being, engagement, and professional identity.

### Perceived demands and resources (RQ1 and RQ2)

The demand–strain pathway of the JD-R model highlights how sustained high demands can lead to strain and exhaustion. In line with this, nurses in the younger age group (27–51 years) reported higher physical strain, greater exhaustion, and a stronger desire for shorter working hours, indicating heavier workload demands early in their careers. In contrast, nurses in the older age group (52–74 years) may draw on accumulated experiential knowledge to organise tasks more efficiently, prioritise effectively, and make greater use of ergonomic or technical solutions, contributing to lower perceived physical strain and fatigue. These patterns align with evidence suggesting that experience and professional self-regulation can buffer the impact of demanding work environments over time (Bakker et al., 2023; Ilmarinen, 2006). Limited recognition reported by nurses in the younger group further points to a potential ERI, which may exacerbate strain and undermine motivation. Gender differences were also evident, with women more likely than men to report psychological strain and higher self-imposed work pressure, reflecting the gendered distribution of emotional labour in healthcare (Purvanova and Muros, 2010). Across age and gender groups, staffing shortages and high work pace were widely reported, consistent with earlier findings identifying work overload, lack of formal rewards, and work–life interference as central job demands in nursing (Broetje et al., 2020). Occupational stress has further been shown to negatively affect safety and productivity, underscoring the broader organisational consequences of sustained high demands (Xue et al., 2025).

The resource–motivation pathway of the JD-R model emphasises how resources support engagement, professional growth, and well-being. Nurses in the older age group more frequently reported that their skills and knowledge were fully utilised, reflecting higher autonomy, a key job resource that typically increases with seniority. Social support from colleagues and managers was generally high across groups, consistent with evidence demonstrating its potential protective role against stress and burnout (Van der Heijden et al., 2020). Female nurses in the younger age group reported a particularly strong sense of social community at work, which may enhance job satisfaction and retention (Broetje et al., 2020; Van der Heijden et al., 2020). However, previous research has also shown that greater autonomy, social support, and participation in decision-making can coexist with heightened perceptions of time pressure and emotional demands (Fernandez de Henestrosa et al., 2023), highlighting the complex and sometimes paradoxical interplay between resources and demands. These differences in perceived demands and resources were reflected in professional identity outcomes. Nurses in the older age group reported greater joy, pride, and meaning in their work, whereas nurses in the younger group facing higher demands and lower recognition may experience challenges in identity development, engagement, and retention. Overall, the interaction between demands and resources appears central to shaping nurses’ well-being and professional identity, with variation across age and gender. This interpretation is consistent with longitudinal evidence demonstrating that job demands negatively affect health outcomes, whereas job resources exert a protective influence on well-being and sickness absence over time (Mäkikangas and Kinnunen, 2003; Thapa et al., 2022). Strengthening key resources – particularly recognition, autonomy, and targeted support for younger nurses – may therefore promote sustainable work practices and professional identity development.

### Age-related differences (RQ3)

Nurses in the older age group reported better overall well-being, more rest between shifts, and fewer signs of emotional exhaustion than their younger counterparts, despite being more likely to report work-related injuries and poorer sleep quality. This pattern may partly reflect a ‘healthy worker effect’, whereby those who remain in nursing at older ages constitute a more resilient subgroup. Previous research has shown that physical demands are associated with disability, whereas emotional demands are linked to burnout among older nurses (Van der Heijden et al., 2020). At the same time, older nurses may benefit from greater professional autonomy, well-developed coping strategies, and stronger role security, all of which can buffer high job demands (Liu et al., 2024). In contrast, nurses in the younger group reported greater difficulties with recovery between shifts and higher emotional exhaustion, potentially reflecting early-career challenges such as steep learning curves, lower autonomy, and less secure professional identity. Family responsibilities, including caring for young children, may further intensify work–life conflict in this group (Carlsson et al., 2009). Importantly, some observed age differences may also reflect cohort effects rather than purely career-stage effects. Experienced nurses were trained and entered the profession in a different era, characterised by distinct educational models, organisational conditions, and societal expectations, which may shape coping strategies and responses to work demands. Supporting this interpretation, generational research has shown that Generation X and Baby Boomers differ from younger nurses in their approach to workplace norms, work–life balance, and task organisation, whereas Generation Z emphasises both work–life separation and autonomy over task execution (Tan and Guey Fong, 2023). Together, these findings suggest that age-related differences may reflect an interplay of career stage and generationally shaped values. This aligns with life-course and work ability perspectives, emphasising the need to balance demands and resources to sustain engagement across the nursing career.

### Gender differences (RQ4)

Female nurses in this study reported greater psychological strain and poorer health outcomes than their male counterparts, consistent with previous research showing higher prevalence rates of post-traumatic stress disorder, anxiety, and depression among women in nursing (Saragih et al., 2021). Male nurses, in contrast, perceived somewhat greater control over their work but reported lower levels of pride and meaning, suggesting potential gender differences in sources of work-related fulfilment and professional identity. These patterns can be understood within the broader context of nursing as a gendered profession, in which women often carry a disproportionate share of emotional, relational, and caregiving demands. From a JD-R perspective, this may increase vulnerability to psychological strain when emotional demands are high and resources insufficient. The ERI model (Siegrist, 1996) offers a complementary explanation, proposing that stress arises when sustained effort is not matched by adequate recognition or reward. This mechanism may be particularly relevant for female nurses in the present study, who reported higher psychological strain despite substantial work contributions. Supporting this interpretation, recent research indicates that female nurses experience higher levels of workplace stress than their male colleagues (Sunitha and Tejashwini, 2024), potentially reflecting gendered expectations, role overload, and inequities in recognition and reward structures.

### Resilience, work engagement, and protective factors

Despite the substantial demands identified, the findings also highlight the resilience of the nursing workforce. Nurses across age and gender groups reported high levels of joy and meaningfulness in their professional roles. Elevated joy at work has been associated with greater work engagement, psychological resilience, and improved clinical performance (Cochran, 2024), whereas resilience more broadly has been shown to reduce burnout and turnover intentions (Correia-Foster, 2023). In the present study, high levels of pride, meaning, and job satisfaction – particularly among older nurses – suggest the presence of strong motivational resources. Nurses’ commitment often derives from the meaningful nature of their work and the satisfaction it provides (Van der Heijden et al., 2020). However, the protective effects of pride and professional identity may be insufficient if organisational demands remain chronically high. According to the JD-R model, when demands consistently outweigh available resources, even highly motivated workers are at increased risk of exhaustion and disengagement. From a sustainability perspective, these findings underscore the importance of strengthening organisational resources, supporting nurses during early career transitions and ensuring adequate recognition for effort, particularly among younger nurses, to promote long-term engagement and retention.

### Healthcare systems, policy, and future research implications (Swedish context)

At a healthcare systems and policy level, the findings indicate that early-career nurse strain reflects structural conditions related to staffing, work organisation, and governance, rather than individual vulnerability. In the Swedish context – characterised by high efficiency demands, persistent staffing shortages, and increasing care complexity – such imbalances between demands, resources, and recognition risk undermining both retention and sustainable working lives. The results align with national policy priorities such as *Good and Close Care* (Ministry of Health and Social Affairs, 2021), aimed at strengthening person-centred and accessible healthcare, long-term competence supply, and systematic work environment management, highlighting the need for workforce strategies that explicitly consider career stage. Policies supporting structured transition-to-practice programmes, protected time for supervision and mentorship, and minimum standards for onboarding and ergonomic working conditions – consistent with regulations from the Swedish Work Environment Authority and European Union strategies for healthy workplaces – may reduce early-career strain and promote longer professional trajectories. Further research should use longitudinal and comparative designs to examine how early-career exposure to imbalanced work conditions influences well-being, professional identity, and retention over time. Studies incorporating organisational and policy-level factors, as well as evaluations of system- and organisation-level interventions, are needed to inform evidence-based strategies for a sustainable nursing workforce.

## Strengths and limitations

This study benefits from a large sample size, enhancing statistical precision. Nonetheless, several limitations should be acknowledged. The cross-sectional design precludes causal inferences, as temporal relationships between variables cannot be established. Voluntary participation may have introduced self-selection bias, potentially affecting representativeness. Furthermore, reliance on self-reported data may increase the risk of common method variance and reporting bias (Podsakoff et al., 2003). The study was theoretically informed by the SwAge model, a comprehensive framework for understanding determinants of a sustainable working life across the life course. However, the SwAge model is a conceptual rather than a psychometrically validated measurement instrument. Although survey items were developed to reflect key domains of the model, formal validation of these items as a unified scale was beyond the scope of this study. The findings should therefore be interpreted as theoretically grounded rather than as derived from a validated SwAge-based instrument.

In addition, responses collected on a four-point Likert scale were dichotomised for analytical purposes. Although this approach facilitated interpretability and model stability, it resulted in loss of information and variability, which may have reduced statistical power and obscured nuanced response patterns (MacCallum et al., 2002). Consequently, observed associations may represent conservative estimates. The use of numerous chi-square tests further increases the risk of Type I error due to multiple comparisons. Although this issue is inherent in studies examining multiple outcomes, the findings should be interpreted with caution, with emphasis placed on consistent patterns rather than isolated statistically significant results. The pronounced gender imbalance – reflecting the nursing workforce – and the relatively high median age (51 years) may further limit generalisability, particularly to male nurses and younger professionals. Finally, as participants were drawn exclusively from the Skane region in southern Sweden, contextual factors may limit external validity. Despite these limitations, the study provides valuable insights into how age and gender intersect with nurses’ work environment, health, and professional identity, offering a solid basis for future longitudinal and intervention-based research.

## Conclusion

This study demonstrates that nurses’ work environment, well-being, and professional identity are shaped by the dynamic interplay between job demands and resources, with important variations across age and gender. Applying the JD-R and ERI frameworks, the findings show that high demands coexist with strong professional pride and meaning, but that insufficient recognition, autonomy, and recovery opportunities particularly challenge younger nurses. Strengthening key resources – especially autonomy, recognition, and social support – while addressing systemic and organisational demands may promote sustainable work practices, support professional identity development, and reduce early-career strain. These findings offer actionable insights for healthcare employers, educators, and policymakers seeking to sustain a resilient, engaged, and age-diverse nursing workforce. Future research should further examine underlying organisational and psychosocial mechanisms and explore the long-term consequences for nurses’ health, retention, and career sustainability through longitudinal designs.

**Key points for policy, practice and research**

• Strengthening core job resources – autonomy, recognition, and social support – is essential to counterbalance high work demands and promote sustainable nursing work environments.
• Early-career nurses represent a structurally vulnerable group and may benefit from targeted transition-to-practice support, including mentorship, ergonomic training, and coping strategies to reduce strain and improve retention.
• Gendered patterns of emotional labour remain evident in nursing, highlighting the need for equitable workload distribution, recognition practices, and organisational policies that address gender-related disparities.

## Supplemental Material

sj-doc-1-jrn-10.1177_17449871261426973 – Supplemental material for Balancing demands and rewards: a cross-sectional examination of well-being and professional identity among nurses Supplemental material, sj-doc-1-jrn-10.1177_17449871261426973 for Balancing demands and rewards: a cross-sectional examination of well-being and professional identity among nurses by Cicilia Nagel and Kerstin Nilsson in Journal of Research in Nursing
